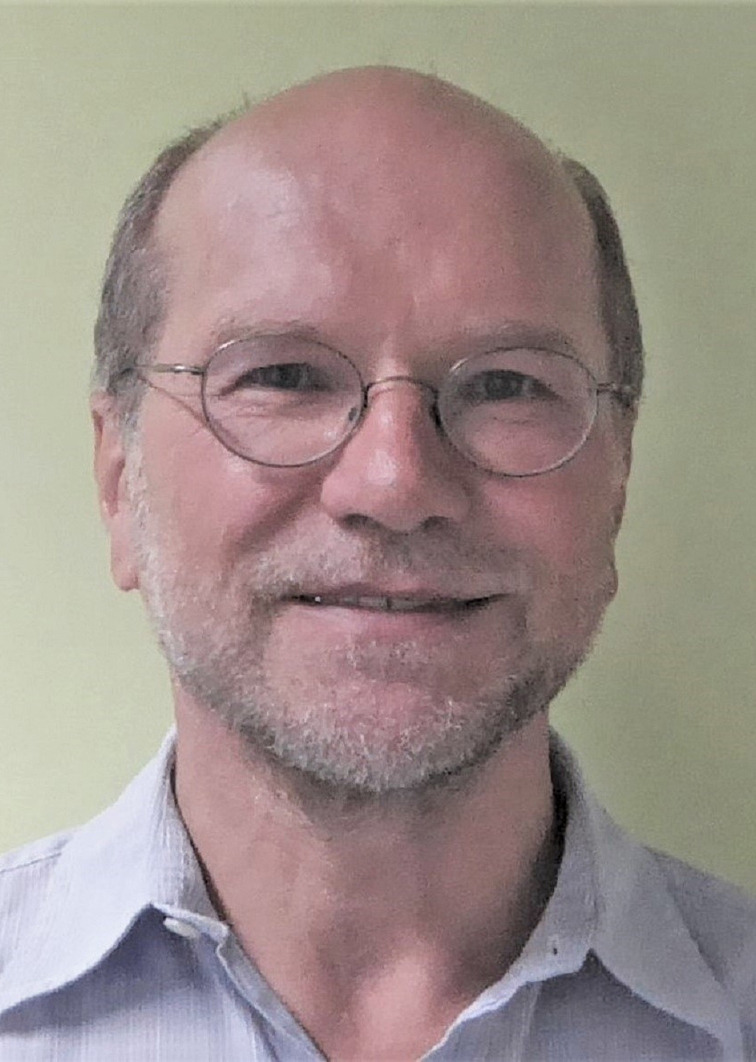# Thermodynamic Stability of G‐Quadruplexes: Impact of Sequence and Environment

**DOI:** 10.1002/cbic.202100127

**Published:** 2021-05-02

**Authors:** Jagannath Jana, Klaus Weisz

**Affiliations:** ^1^ Institute of Biochemistry Universität Greifswald Felix-Hausdorff Str. 4 17489 Greifswald Germany

**Keywords:** calorimetry, DNA, G-quadruplexes, thermodynamics, topology

## Abstract

G‐quadruplexes have attracted growing interest in recent years due to their occurrence *in vivo* and their possible biological functions. In addition to being promising targets for drug design, these four‐stranded nucleic acid structures have also been recognized as versatile tools for various technological applications. Whereas a large number of studies have yielded insight into their remarkable structural diversity, our current knowledge on G‐quadruplex stabilities as a function of sequence and environmental factors only gradually emerges with an expanding collection of thermodynamic data. This minireview provides an overview of general rules that may be used to better evaluate quadruplex thermodynamic stabilities but also discusses present challenges in predicting most stable folds for a given sequence and environment.

## Graphical abstract

**Biological functions** of G‐quadruplexes and their properties as exploited in various technological applications depend on their major fold adopted under specific conditions. The present minireview guides through structural and environmental factors that have been identified to play a major role in the formation and stability of G‐quadruplexes.

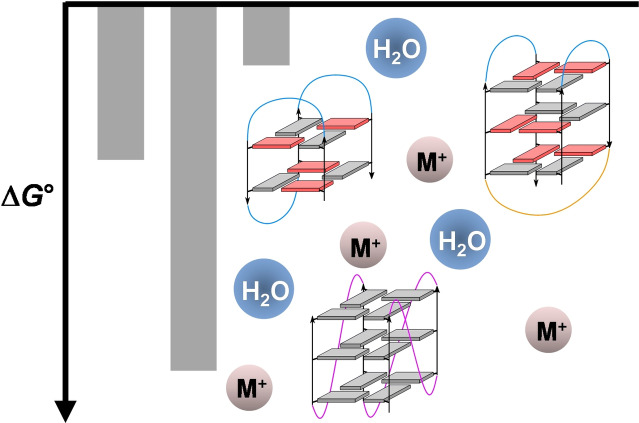

## Introduction

G‐quadruplex (G4) structure and stability has been scrutinized for more than two decades, owing to the growing interest for these nucleic acid secondary structures in biology, medicine, as well as in bio‐ and nanotechnology.[^1^, ^2^, ^3^] Numerous studies have contributed to a better understanding of folding pathways for G‐rich sequences and have significantly expanded the topological and conformational landscape of these remarkably diverse structures. With growing structural information at hand, stabilities of G‐quadruplexes have also increasingly been assessed as a function of particular topologies, loop lengths, overhang sequences, and the outer environment. First attempts to predict stabilities of G‐quadruplexes have been made[^4^, ^5^] but knowing structure‐stability relationships in more detail will be essential for a better understanding of G4 folding pathways.

Observation of a most stable G4 conformer as major species requires reversible conditions under thermodynamic control. However, energy barriers for G4 folding and unfolding are relatively high with Δ*G* ^#^∼15 k*T*, resulting in slow folding but also allowing kinetic trapping of folded G4 species.[^6^, ^7^] Consequently, both kinetic and thermodynamic studies are necessary for a comprehensive description of quadruplex folding.[^8^, ^9^, ^10^] Whereas valuable kinetic studies based on spectroscopic and modeling approaches have increasingly been reported on folding pathways in the more recent past,[^6^, ^11^, ^12^, ^13^] this minireview summarizes our current understanding of G‐quadruplex stability as a function of the G‐rich sequence and the outer environment.

### Methodologies for evaluating G‐quadruplex stability

Evaluating G‐quadruplex stabilities has been approached in various ways.[^14^, ^15^, ^16^, ^17^] These include a simple determination of G‐quadruplex melting temperatures *T*_m_, i. e., the temperature at which 50 % of the quadruplex has unfolded (melted). Various spectroscopic methods such as fluorescence, circular dichroism, or NMR can be employed but measuring temperature dependent UV absorbances is most frequently used for nucleic acid structures and often common practice for novel quadruplex structures. *T*_m_ values give an indication of the G4 thermal stability under the given solution conditions. Although melting temperatures in many cases show a reasonably good correlation with Gibbs free energies at physiological temperatures, relative thermodynamic stabilities at a given temperature far from melting may significantly vary from the relative order of melting temperatures due to different temperature dependencies of equilibrium constants and Gibbs free energies.

For intramolecularly folded quadruplexes, *K*_fold_=1 and Δ*G*°=0 at *T*_m_ and extrapolation to other temperatures requires a more comprehensive thermodynamic profile with known enthalpic and entropic contributions Δ*H*° and Δ*S*°. In general, these can be extracted from a melting curve after proper pre‐ and post‐transitional baseline corrections, providing populations of folded and unfolded species and therefore equilibrium constants as a function of temperature. This allows for a subsequent van't Hoff analysis to yield both Δ*H*° and Δ*S*° and thus a complete thermodynamic signature of the (un)folding process. However, due to inherent limitations of a van't Hoff analysis, results may often be questionable. Thus, heating/cooling rates must be slow enough for the system to always be in thermodynamic equilibrium. Also, analysis relies on a simple two‐state process but many pathways have been shown to proceed through multiple intermediate states.[^18^, ^19^] Finally, a van't Hoff analysis is mostly based on a linear plot ln *K*_fold_=f(1/*T*). This linearity assumes a negligible temperature dependence of standard enthalpies and entropies, i. e., Δ*C*_p_ *°*=0. If changes in molar heat capacity Δ*C*_p_ *°*≠0 as expected for quadruplex folding,[^20^, ^21^, ^22^] small curvatures of the van't Hoff plot will result. However, a non‐linear fit within the limited temperature range accessible for biological macromolecules will foreseeably fail to allow for a reliable determination of Δ*C*_p_°.

Finally, calorimetric methods like differential scanning calorimetry (DSC) directly measure heat effects upon melting and should thus be the method of choice for evaluating the folding thermodynamics.^[23]^ In addition to a van't Hoff analysis to yield a Δ*H*°_vH_, a model‐independent determination of a calorimetric enthalpy Δ*H*°_cal_ is not strictly limited to a two‐state transition but also includes possible intermediates during the (un)folding process. As an added advantage, ratios of Δ*H*°_vH_/Δ*H*°_cal_ may give valuable information on the presence of intermediates for ratios <1 or aggregation and cooperativity effects for ratios >1. Unfortunately, DSC experiments and their analysis are also prone to general limitations and uncertainties. Although heat capacities are directly measured in a DSC experiment, Δ*C*_p_ *°* can rarely be accessed in a reliable way due to problems of a proper baseline correction, blurring the small Δ*C*_p_ *°* effects for G‐quadruplex formation.^[24]^ Also, care must be exercised when determining the quadruplex concentration because any errors in concentration will directly translate into errors of Δ*H*°_cal_. This may be a major problem if other species coexist but remain unnoticed as for the formation of high‐melting multimers.

### G‐quadruplex structures and their thermodynamic stability

**Figure 1:**
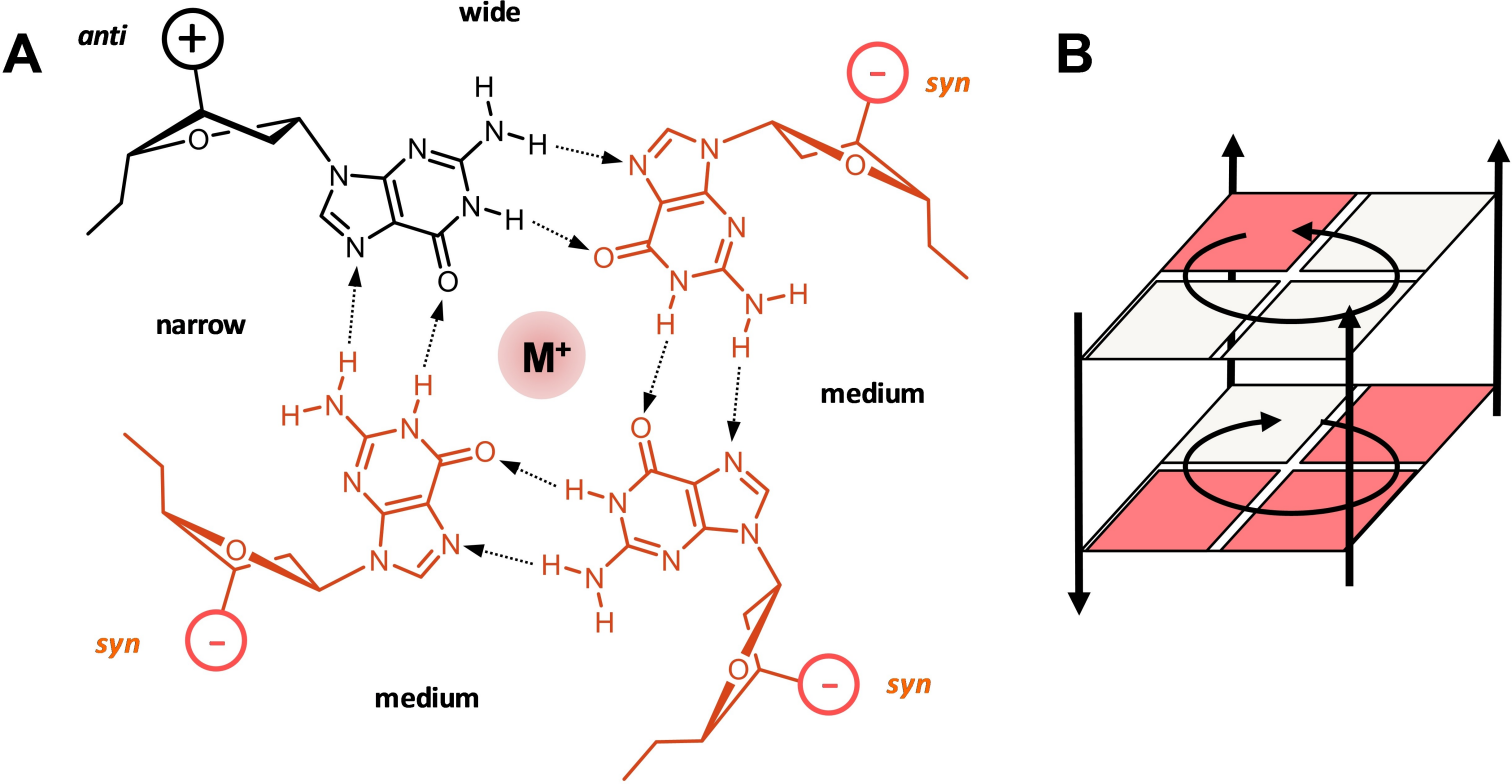
(A) Planar arrangement of four guanine nucleotides with a central metal ion to form a G‐tetrad. The *anti*‐ and *syn*‐Gs are indicated by black and red colors, respectively; the tetrad polarity is determined by the direction of hydrogen bonds with medium, narrow, and wide grooves indicated. (B) Two stacked G‐tetrads of opposite polarity; guanine bases are represented by squares. The *syn*‐*anti* pattern in (A) and (B) represent just one example of various possibilities.

**Figure 2:**
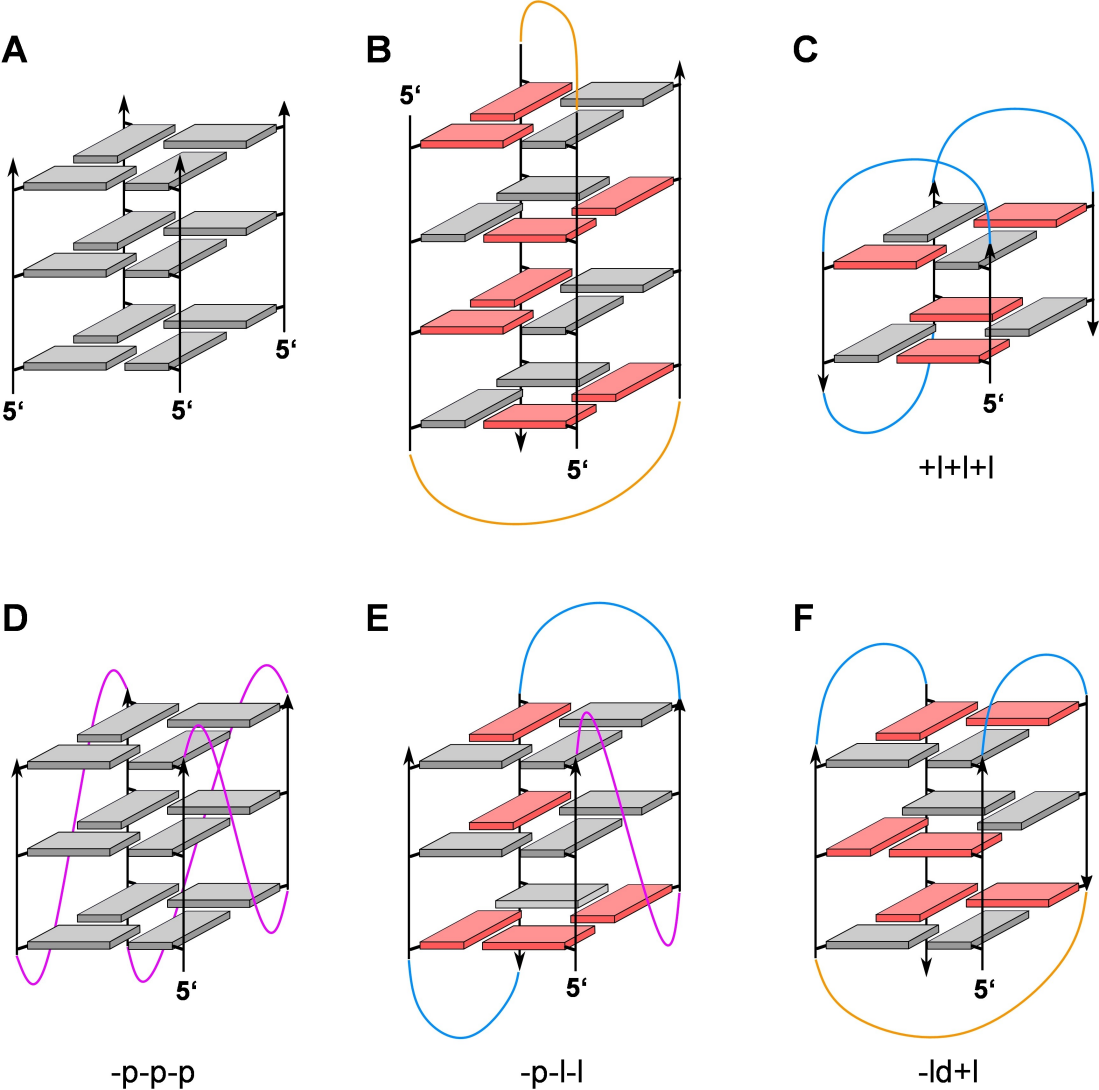
Representative G‐quadruplex topologies; *anti*‐ and *syn*‐guanosines of the G‐core are characterized by grey and red rectangles, respectively; propeller, lateral, and diagonal loops are colored magenta, blue, and orange. (A) Tetramolecular G4 with all strands running parallel and all Gs adopting an *anti*‐conformation. (B) Four‐layered bimolecular G4 as adopted by d(G_4_T_4_G_4_) in the *Oxytricha nova* telomere sequence; two diagonal loops connect pairs of antiparallel G‐tracts. (C) Antiparallel ‘chair’‐type topology +l+l+l with two stacked G‐tetrads and three lateral loops running in a clockwise direction as found for the thrombin binding aptamer (TBA) sequence. (D) Monomolecular parallel G4 with three propeller loops progressing in an anti‐clockwise direction (−p−p−p). (E) (3+1)‐Hybrid (hybrid‐1) topology with one propeller and two lateral loops running anti‐clockwise (−p−l−l). (F) Antiparallel ‘basket’‐type topology ‐ld+l with one central diagonal and two lateral loops.

Canonical G‐quadruplexes are composed of a helical stack of 2–4 G‐tetrads that are formed by a square planar arrangement of four guanine bases held together by a cyclic array of eight Hoogsteen hydrogen bonds (Figure 1). Coordination of metal ions within the central cavity of the G‐core stabilizes these tetra‐stranded architectures to make them competitive nucleic acid secondary structures under various conditions. Although tetramolecular and bimolecular quadruplexes have extensively been studied, monomolecular scaffolds have received most attention due to their high relevance in biological systems. Here, the four G‐columns are connected by intervening sequences that either bridge two adjacent parallel strands, two adjacent antiparallel strands, or two distal edges of an outer G‐tetrad to form a propeller, lateral, or diagonal loop, respectively (Figure 2). Canonical G‐quadruplexes with non‐interrupted G‐columns can be of a parallel, antiparallel, or (3+1) hybrid‐type topology. Because topologies are tightly linked to the loop progression, the structural G4 diversity is best described by the sequential arrangement of connecting loops. Thus, propeller, lateral, and diagonal loops are represented by “p”, “l”, and “d” and their progression is described in relation to a proposed frame of reference with the 5′‐terminus at the lower right corner of the G‐core.[^25^, ^26^] Clockwise and anti‐clockwise progression of propeller and lateral loops are denoted by a preceding “+” or “−“, giving a simple descriptor such as “−p−l−l” for a (3+1) or hybrid‐1 topology (Figure 2E). Of note, such a simple description of quadruplex structure does not include information on the number of G‐tetrads and the geometry of grooves, nor does it discriminate among different patterns of glycosidic bond angles along the individual G‐columns. The latter determines the polarity of stacked tetrads and gives rise to homopolar or heteropolar stacking interactions (Figure 1B).

Knowing the thermodynamics of G‐quadruplex formation is the key to predicting major folds of G‐rich sequences, prerequisite for a better understanding of their interactions in a given environment. Formation and stacking of G‐tetrads constitutes the main driving force for folding into these four‐stranded nucleic acid structures. This is associated with the coordination of cations between the G‐quartets.^[27]^ Early calorimetric studies on the formation of tetramolecular architectures devoid of loops and long overhang sequences are expected to approximate basal secondary interactions within an unrestrained G‐core. Thus, irrespective of sodium or potassium ions in the buffer, an enthalpy Δ*H*°∼−80 kJ/mol per tetrad has been reported for quadruplexes composed of four d(TG_3,4_T) oligonucleotides.[^28^, ^29^] On the other hand, subtracting the thermodynamic folding profile of a two‐layered from that of a three‐layered DNA aptamer quadruplex comprising identical loops yielded a Δ*G*°_20_ of −9.2 kJ/mol and a Δ*H*° of −61 kJ/mol.^[30]^ Given the prominent role of tetrad stacking in G4 stabilization, it is noteworthy that two‐layered quadruplexes with only two stacked G‐tetrads are frequently observed and may even compete with three‐layered quadruplexes. Here, additional tertiary interactions through the formation of stacked dimers or the stacking of loop and overhang sequences are mostly responsible for their often remarkable thermodynamic stability.^[9][31]^ Interestingly, whereas the formation of a two‐tetrad unit with a sandwiched K^+^ ion is strongly enthalpy‐driven, the formation of a quartet‐K^+^‐triplet unit as featured by slip‐stranded two‐tetrad quadruplexes was found to be entropically favored.^[27]^ As a result of such a positive *T*Δ*S*° contribution, the latter is anticipated to become more populated at higher temperatures.

Tetramolecular constructs associate to preferably form parallel quadruplexes with all four strands pointing into the same direction and with all G residues adopting a more favorable *anti* glycosidic torsion angle. Consequently, tetramolecular G‐quadruplexes with an antiparallel strand arrangement and/or *syn* conformations must usually be enforced by selective substitutions with *syn*‐favoring G analogs such as 8‐bromo‐ or 8‐methyl‐guanosine.[^32^, ^33^, ^34^] For intramolecular quadruplexes, these observations also suggest an intrinsically more favorable parallel G‐core. Apparently, intervening sequences linking the G‐columns of the quadruplex core often exercise significant influence on the formation of parallel, antiparallel, or hybrid‐type topologies. In fact, besides steric effects, loops are often engaged in additional tertiary interactions that strongly depend on the type of loop, making them major determinants of quadruplex folding. It has even been claimed that loop interactions in a non‐parallel human telomeric G4 contribute more to the G‐quadruplex stability than G‐tetrad stacking.^[35]^

### Impact of loop length

Topologies of intramolecular G‐quadruplexes are directly tied to the progression of loops that link their four G‐columns. Whereas G‐tracts of a parallel G4 are connected by three propeller loops, lateral/diagonal loops link two adjacent/distal G‐columns with opposite 5’‐3’‐backbone orientation to form antiparallel or (3+1) hybrid‐type structures (Figure 2). Based solely on its geometry, an intervening sequence with a single nucleotide can easily form a one‐nucleotide (1‐nt) propeller loop. In contrast, lateral loops bridging a narrow or wide groove generally comprise >1 or >2 nucleotides, respectively, and a diagonal loop bridging distal edges of a G‐tetrad is expected to require >3 nucleotides. However, molecular modeling studies but also experimental observations even suggest toleration of lateral and diagonal loops as short as one and three nucleotides, respectively.[^36^, ^37^, ^38^] It should be noted that steric constraints on the loop as mentioned above strictly apply to three‐layered quadruplexes. Altering the number of helically stacked tetrads may affect geometric requirements, in particular for propeller loops linking the two faces of the G‐quadruplex core.^[38]^

Going beyond these inherent mechanical limitations exerted by a loop, the influence of loop lengths on the stability and topology of intramolecular G‐quadruplexes has extensively been studied during the past two decades. General findings reported on intramolecular quadruplexes in a K^+^ buffer can be summarized as follows: (i) An increase in loop length is associated with a gradual decrease in the thermodynamic stability.[^39^, ^40^, ^41^, ^42^] Thus, the melting temperature of a parallel quadruplex d(G_3_L_i_G_3_L_j_G_3_L_k_G_3_), exclusively comprising 1‐nt loops with randomized sequences L_n_=A, T, or C, decreased by 8 °C and 16 °C in a 20 mM K^+^ buffer solution when elongating the third loop by one and two nucleotides, respectively.^[41]^ This was associated with a loss in Δ*G*° of about 8 and 12 kJ/mol at 37 °C as determined by a van’ Hoff analysis of UV melting. It should be mentioned, however, that G4 thermal and thermodynamic stabilities may noticeably differ depending on the ionic strength and in particular on the potassium cation concentration.[^43^, ^44^] (ii) With a further increase in total loop length, G4 stabilities tend to level off and there is also a growing tendency of G‐rich sequences to fold into antiparallel or hybrid‐type topologies.[^41^, ^45^] These results suggest higher thermodynamic stabilities with longer loops for antiparallel quadruplexes and weaker correlations between loop length and stability when compared to parallel topologies. Of note, additional higher‐order G4 aggregates including dimers and trimers have been reported to more frequently form from sequences comprising short loops.^[45]^ (iii) A single‐nucleotide intervening sequence seems special in only accommodating a propeller loop through its geometric constraints but also in imparting high thermodynamic stability to parallel quadruplexes.[^40^, ^41^]

**Figure 3:**
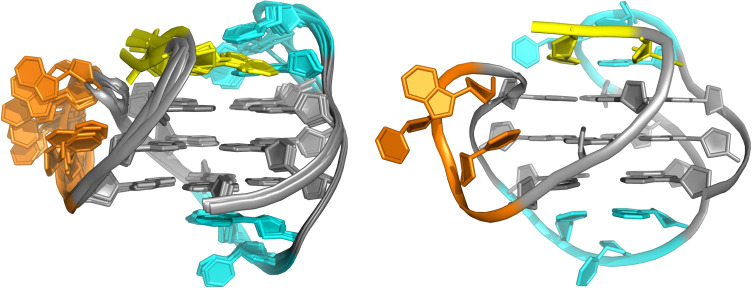
Representative (right) and ten superimposed NMR high‐resolution structures (left) of a hybrid‐1 (−p−l−l) quadruplex formed by a human telomeric sequence in K^+^ solution (PDB 2JSK).^[46]^ G‐core, 5’‐overhang, propeller, and lateral loops are colored grey, yellow, orange, and cyan, respectively. Whereas only minimal interactions are observed for bases within the flexible 3‐nt propeller loop, bases of the well‐defined and better structured 3‐nt lateral loops form a base triad with the 5’‐TA overhang (top) and a Hoogsteen AT base pair (bottom) stacked onto the outer G‐tetrads.

Parallel quadruplexes and thus propeller loops have been suggested to be more favored by entropic contributions.^[36]^ In fact, propeller loops positioned at the sides of a G‐core and thus lacking more intimate interactions with outer tetrads exhibit higher flexibility in most high‐resolution NMR structures with only rather weak and short‐lived interactions of propeller loop residues (Figure 3). Based on thermodynamic profiles for parallel G‐quadruplex formation, a considerable drop of favorable enthalpies Δ*H*° as found with an increase of total loop length is only partially compensated by more favorable entropic contributions Δ*S*°.[^41^, ^44^] This may point to distortions of the G‐core geometry induced by long flexible loops. Additionally, solvent exposed bases within a propeller loop may be partially stacked in a single‐stranded state with an enthalpic penalty for unstacking prior to G4 folding.

In contrast, lateral and diagonal loops of non‐parallel structures are positioned on top of outer tetrads and are expected to benefit from significant enthalpic contributions, e. g. through base stacking onto the outer G‐tetrad or hydrogen bonding to form capping base pairs and base triads (Figure 3). Increasing the length of intervening sequences to allow for the formation of lateral/diagonal loops, these additional tertiary interactions may ultimately outcompete the preference for parallel topologies. Because a longer lateral or diagonal loop may enable a growing number of tertiary interactions, favorable enthalpic contributions in antiparallel and hybrid‐type structures may mask lower stabilities generally associated with longer loops. Thus, DSC studies on two‐layered thrombin binding aptamer variants that fold into a chair conformation demonstrate significant stabilization of the quadruplex structure due to favorable enthalpic contributions of the different loops ranging from −35 kJ/mol<Δ*H*°_cal_<95 kJ/mol in a 100 mM K^+^ buffer.[^30^, ^47^]

Clearly, the latter thoughts on the impact of additional interactions involving loop but also overhang sequences set limits to a straightforward prediction of quadruplex stabilities based solely on loop lengths. Such tertiary interactions are strongly sequence‐dependent and often involve other domains non‐adjacent in the primary sequence, restricting reliable predictions of their outcome. In fact, except for unstructured propeller loops with only weak interactions, correlations between the total loop length and thermodynamic stabilities are superimposed by additional enthalpic contributions of different magnitude from tertiary interactions in antiparallel or hybrid structures. Correspondingly, no significant correlation was observed in a comprehensive thermodynamic evaluation of more than 30 naturally occurring intramolecular DNA quadruplexes exhibiting diverse loop lengths, loop compositions, and formed topologies with thermodynamic stabilities ranging from −7 kJ/mol≤Δ*G*°_37_≤−65 kJ/mol in the presence of 100 mM K^+^.^[48]^

**Figure 4:**
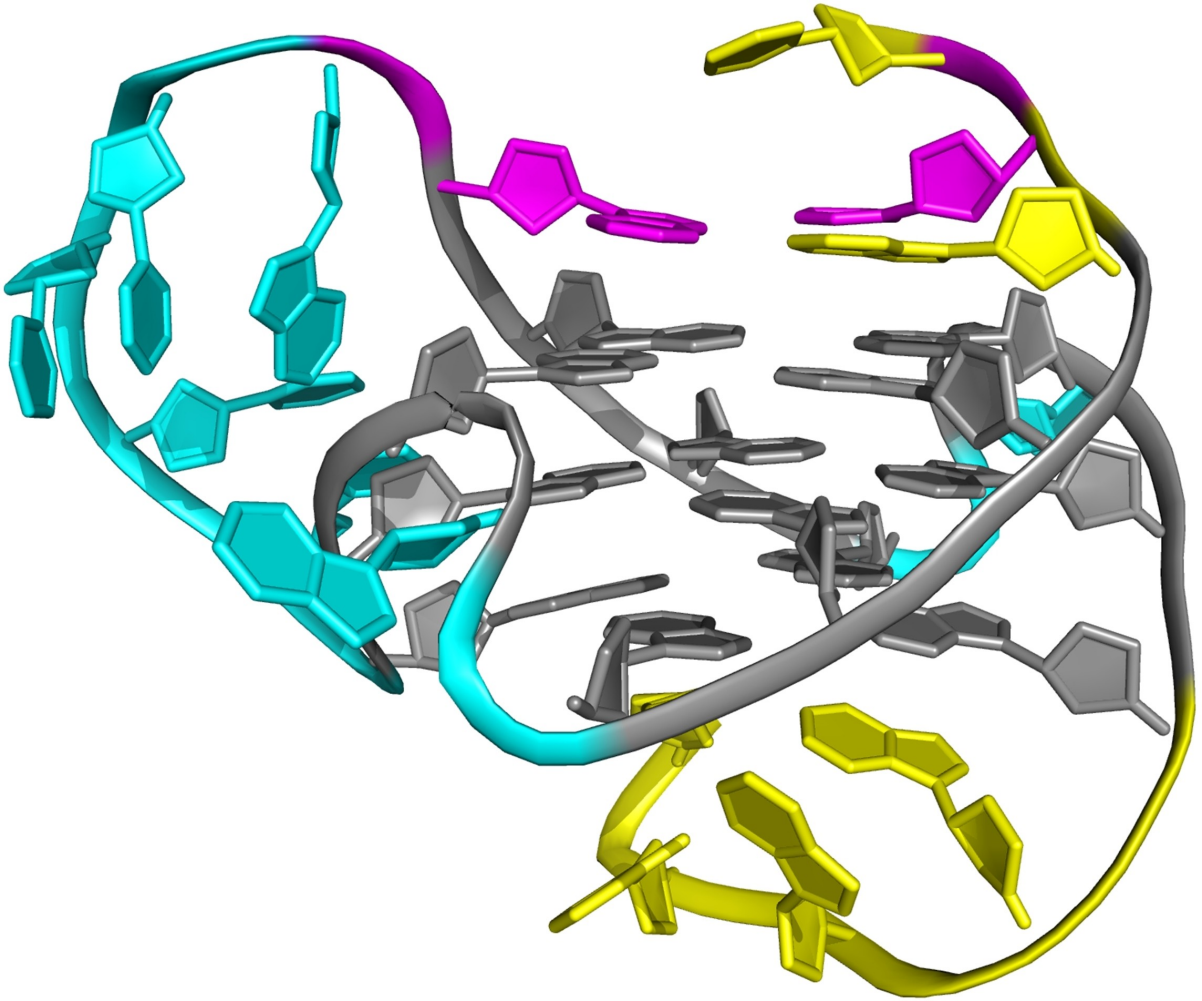
NMR high‐resolution structure of a parallel quadruplex (−p−p−p) formed by a *c‐myc* promoter sequence (PDB 6NEB).^[49]^ An adenine base at the 3’‐end of the long 6‐nt central propeller loop is Hoogsteen hydrogen‐bonded to a 5’‐flanking T residue (both shown in magenta) and stacks as part of an ATG triad onto the outer G‐tetrad; G‐core, overhang sequences and propeller loops are colored grey, yellow, and cyan, respectively.

It should also be mentioned that an anticipated enthalpy‐mediated folding into lateral/diagonal loops to form non‐parallel structures may only poorly apply in case of very long loops. Here, more stable base‐base interactions can even be formed within a propeller loop and long propeller loops of ≥6 residues have been reported to be well tolerated if engaged in stacking or hydrogen bond interactions (Figure 4).[^49^, ^50^, ^51^, ^52^] Accordingly, a 9‐nt central propeller loop in a G‐quadruplex of the human CEB25 minisatellite was shown to be anchored to the 5′‐overhang by specific base pairings, significantly contributing to the G4 stability with an increase in enthalpy reported to be about 70 kJ/mol.^[50]^

RNA quadruplexes with thermodynamic stabilities being generally higher when compared to their DNA counterparts are known for their strong propensity for parallel topologies that only feature *anti*‐residues and medium grooves for their G‐core. This may be explained by the preference of ribonucleotides to adopt an *anti*‐conformation but has also at least partially been attributed to (i) the replacement of thymines by less hydrophobic uracil bases and (ii) to unfavorable effects of 2’−OH substituents when situated in a quadruplex narrow groove.[^22^, ^53^, ^54^] Thus, additional loop interactions in RNA quadruplexes are mostly insufficient to overcome the much higher energy gap between parallel and antiparallel topologies. As a consequence of their loop independent parallel fold, a strong correlation up to longer loops has been observed between total loop length and decreasing RNA G4 stabilities.^[55]^

### Impact of loop composition

As outlined above, lateral and diagonal loops are more prone to additional stabilizing interactions compared to propeller loops. These may include stacking interactions within the loop or onto a neighboring G‐quartet but also base pairing between loop and overhang residues. As a consequence, antiparallel quadruplexes are anticipated to strongly benefit from enthalpic contributions. This is supported by thermodynamic studies on a telomere sequence of *Oxytricha nova* d(G_4_‐L‐G_4_) with loop L consisting of either four thymine residues T_4_ or of non‐nucleosidic linkers, namely (1’‐2’‐dideoxyribose)_4_, (propanediol)_4_, and hexaethylene glycol residues. The wild‐type oligonucleotide d(G_4_T_4_G_4_) has been shown to form a dimeric G4 comprising four G‐tetrads and two diagonal T_4_ loops that link distal edges on either side of the G‐core (Figure 2B). Conserving this fold‐back structure for the modified species with non‐nucleosidic linkers in the presence of Na^+^, the latter showed a noticeable decrease in stability with a less negative Δ*H*° attributable to the absence of H‐bond and stacking interactions of loop bases.^[56]^ In another report on the use of non‐nucleosidic loops, substituting propanediol, octanediol, and hexaethylene glycol for all three TTA loops in a human telomeric sequence resulted in characteristic changes of CD signatures. These observations strongly suggested conversion of the native antiparallel/hybrid‐type fold in a sodium/potassium buffer solution into a parallel quadruplex as a consequence of lacking stabilizing interactions within a non‐nucleosidic lateral/diagonal loop.^[57]^ In contrast, an increase in stability was reported if single loop nucleotides L in a parallel‐stranded quadruplex d(TG_3_LG_3_LG_3_LG_3_T) were replaced by abasic 1’2’‐dideoxyribose.^[58]^ Apparently, lacking a solvent‐exposed hydrophobic nucleobase seems beneficial in case of a short propeller loop without additional base interactions.

There are several reports on subtle sequence effects on the stability of intramolecular quadruplexes.[^58^, ^59^, ^60^, ^61^] A clear trend correlating sequence with G4 stability is hampered by tertiary interactions in case of longer loops but also by cation dependencies and topological changes in sequence variants. However, a more general rule relates to the position‐dependent effect of loop adenines. Thus, each substitution of a single adenine for thymine in a 1‐nt propeller loop lowered G4 melting by ∼6–8 °C.[^58^, ^60^, ^61^] Also, in contrast to a stabilizing adenine residue located at the 3’‐position of 2‐nt and 3‐nt lateral loops, a decrease of melting temperature was generally observed with a 5’‐loop adenine.[^21^, ^59^, ^62^, ^63^] These destabilizing effects may indicate penalties due to a larger solvent‐exposed hydrophobic surface area of the purine base with restricted stacking interactions in 1‐nt loops or as a first loop residue. On the other hand, a less negative Gibbs free energy upon G4 formation may again result from a partially stacked adenine base in the unfolded single‐stranded state. Such an adenine‐dependent destabilizing effect may also bear biological relevance by selecting for major G4 loop isomers with A‐deficient propeller loops in promoter sequences of oncogenes such as *c‐myc*.[^44^, ^61^] Likewise, telomeric repeats with intervening sequences lacking an adenosine at the first position as observed across different species may have been naturally selected based on G4 stabilities.

### Impact of loop length distribution

In addition to the length and composition of loops, their distribution in intramolecular quadruplexes may also have a profound impact on the conformation and stability of G‐quadruplexes. A comprehensive study on 99 sequences with a different sequential order of their three T_n_ loops has recently been reported and showed significant effects of loop permutations on G4 folding with differences in quadruplex melting by up to 17 °C.^[64]^ Also, quadruplexes with a long central loop were shown to have a higher propensity of folding into an antiparallel or hybrid‐type structure whereas quadruplexes with a short central loop favored a parallel topology. Of note, only thymine‐containing loops were employed in order to minimize contributions from specific interactions involving loop residues and to obtain dependencies mostly due to loop‐mediated geometric restraints imparted to the overall quadruplex structure.

Interestingly, the presence of two 1‐nt loops seems to impose a parallel fold irrespective of the length of the remaining loop.[^41^, ^49^] This may be attributed to a preorganization of fast folding intermediates, constraining a third loop into a propeller‐type conformation. Likewise, a favored parallel fold is frequently observed in the presence of a 1‐nt central loop even in case of two additional longer loops.^[65]^ It is instructive to look at a fragment‐based molecular modeling study. The latter suggested 14 mechanically feasible topologies for three‐layered G‐quadruplexes with non‐interrupted G‐tracts.^[66]^ Looking at possible quadruplex folds encompassing two propeller loops, topologies include three non‐parallel G4 structures, i. e., −pd+p, +l+p+p, and −p−p−l. However, these non‐parallel folds have either not yet been experimentally verified (−pd+p, −p−p−l) or could only be enforced by complementary overhang sequences combined with selective substitutions of *syn*‐favoring G analogs (+l+p+p).^[67]^ On the other hand, two additional and sterically feasible non‐parallel topologies (d+pd and +l+p+l) comprise a single 1‐nt propeller loop in their central position. Whereas a d+pd fold in a Na^+^ buffer has been reported for a two‐layered quadruplex^[68]^ and recently also verified by rational design for a three‐tetrad G4,^[37]^ a +l+p+l topology has only been shown to form as a single major species upon introducing *syn*‐G modifications at specific positions.^[69]^ Consequently, given a 1‐nt loop to only accommodate a propeller‐type conformation, the presence of two 1‐nt intervening sequences and in many cases of a single central 1‐nt loop will possibly favor folding into an all‐parallel G‐quadruplex in K^+^ solutions due to low thermodynamic stabilities and/or slow folding kinetics of competitive folds.

### Impact of outer conditions

Stabilities and reversible transitions of quadruplex structures not only depend on sequence but also on temperature, the presence of different cations, and on water activity. Global fitting of calorimetric and spectroscopic data measured under various conditions to obtain a set of parameters based on a model mechanism has recently been shown to constitute a powerful approach for a comprehensive thermodynamic characterization.[^70^, ^71^, ^72^] It enabled the construction of phase diagrams for human telomeric oligonucleotides, allowing to trace pathways of (un)folding and interconversions for a wide range of outer conditions.

Potassium and sodium cations stabilize G‐quadruplexes by their binding within the central channel of the G‐core, reducing the repulsion of guanine carbonyl oxygen atoms. Upon increasing cation concentration, a typically observed stability enhancement of quadruplexes may result from charge screening effects but can primarily be attributed to cations acting as specifically bound ligands.[^27^, ^39^, ^73^] Microcalorimetric studies on the G4 formation of *c‐myc* sequence variants with a parallel three‐tetrad G4 topology demonstrated a significantly more favorable enthalpic contribution with only partial compensation by a higher entropic penalty when increasing the K^+^ ion concentration.^[44]^ Because the Δ*H°* term to the Gibbs free energy seems to predominate even at higher temperatures, the overall thermodynamic stability of the quadruplexes will usually increase in the accessible temperature range at higher K^+^ concentration.

Generally, the order of overall affinity in water follows K^+^>Na^+^, i. e., K^+^ ions impart more stability to G‐quadruplexes when compared to Na^+^ ions.[^45^, ^60^] A notable exception was reported for a higher‐order telomeric G‐quadruplex, being more stable in sodium than in potassium at physiological temperatures due to more stabilizing interactions between the two G4 units in the sodium conformation.^[74]^ Employing computational methods, a better stabilization of monomeric guanine quadruplexes through K^+^ was shown to almost equally depend on desolvation effects and on the size of the alkali metal cation.^[75]^ For a *c‐myc* sequence exclusively folding into a parallel topology, replacing a 120 mM Na^+^ by a 120 mM K^+^ buffer stabilized the quadruplex by considerably increasing the exothermicity of folding. Despite being compromised by a modest increase in entropic penalty, a significant ΔΔ*G*° at 37 °C of −30 kJ/mol was determined.^[44]^ However, replacing potassium by sodium ions is often found to promote a Na^+^‐induced refolding of a parallel quadruplex into an antiparallel or (3+1) hybrid G4, often challenging a direct assessment of a cation‐specific quadruplex stabilization.^[8]^ Accordingly, three‐layered G4 structures with an antiparallel topology seem to be strongly disfavored in a potassium buffer and there is only one report of an antiparallel chair‐type G4 formed by a human telomeric variant in K^+^ solution.^[76]^ The preferential formation of non‐parallel G4 structures by sodium ions has been attributed to a favored Na^+^ coordination at lateral/diagonal loops.[^45^, ^77^] To assess the impact of cations on an antiparallel topology, a human telomere sequence was fixed to a basket‐type fold through selective substitutions with 2’‐deoxyxanthosine and 8‐oxo‐2’‐deoxyguanosine. Indeed, a rather small but selective stabilization of the antiparallel conformation by Na^+^ with a ΔΔ*G*°_37_ of about −3 kJ/mol when going from a 120 mM K^+^ to a 120 mM Na^+^ buffer was reported.^[78]^

**Figure 5:**
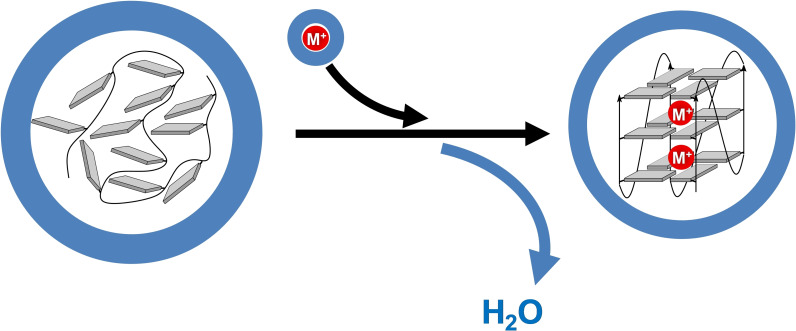
Folding of a G‐rich sequence into a G‐quadruplex in the presence of a solvated cation (red circle) is accompanied by a net release of water; blue spheres represent bound water molecules.

Co‐solutes like ethylene glycol and polyethylene glycol (PEG) decrease water activity and are commonly used as molecular crowding agents to mimic conditions in a cellular environment. Addition of 30 % ethylene glycol enhanced thermal as well as thermodynamic stabilities for d(G_3_T_i_G_3_T_j_G_3_T_k_G_3_) sequences comprising T_n_‐loops ranging from one to five nucleotides in a 100 mM K^+^ buffer solution irrespective of their particular fold.^[65]^ G‐quadruplex stabilization under molecular crowding can be attributed to an osmotic pressure effect due to a net release of water upon quadruplex formation (Figure 5).[^30^, ^47^, ^79^, ^80^] In fact, dehydration has been shown to contribute a favorable hydrophobic component to the Gibbs free energy of G4 folding and to be responsible for a negative change in heat capacity, correlated with the solvent accessible surface area in analogy to protein folding.^[81]^ The overall number of released water molecules seems to depend on the type and composition of loops but also on the metal ion.[^47^, ^78^] Thus, the co‐solute PEG 200 exerted different stabilizing effects to an antiparallel basket‐type G‐quadruplex in Na^+^ and K^+^ buffers, attributable to a differential water release even when folding into the same topology.^[78]^ Likewise, the transition from a parallel to a hybrid‐type fold for a human telomere fragment was found to be driven by a large hydrophobic component through the release of water molecules, assuming a less compact parallel conformation with a larger water‐exposed hydrophobic surface area.^[22]^ As a consequence of variable structure‐ and cation‐mediated effects on the water release/uptake in quadruplex folding, the differential impact of molecular crowding may shift equilibria between G4 species in case of only small differences in their Gibbs free energy.[^82^, ^83^] However, the conversion of a human telomere quadruplex from a hybrid to a parallel topology in K^+^‐containing solution by high molecular weight PEGs was suggested to not depend on crowding effects but to result from conformational selection through PEG, specifically binding a parallel fold with a concomitant reduction of its water‐exposed hydrophobic surface area.[^22^, ^84^]

Major contributors to quadruplex folding and stability as discussed in the preceding sections are summarized in Table 1. Their general effect on G‐rich sequences is also indicated but may vary depending on the particular sequence context.

**Table 1:** Contributing factors to G‐quadruplex stability and their typical impact.

Parameter	Impact on structure and stability
loop length	with longer loops decrease in stability for parallel quadruplexes but also growing preference for the formation of non‐parallel topologies
	
loop composition	destabilizing effect of adenosine in 1‐nt loops or in the 5‘‐position of short loops when compared to T and C residues
	
loop length distribution	two 1‐nt loops but also a 1‐nt central loop favor parallel topologies
	
cation	sodium ions confer less stability than potassium ions and promote formation of non‐parallel topologies
	
molecular crowding	enhanced stability and a tendency of promoting transition into a parallel topology

## Summary and Outlook

An important rationale for the determination of thermodynamic stabilities of G‐quadruplexes and their thermodynamic profiles upon folding derives from efforts to predict a major, i. e., thermodynamically most stable G4 topology of a given G‐rich sequence. Identifying major folds will not only support G4 functional studies in the cellular environment but will also contribute to their targeting in fighting pathological disorders or to their use in technological applications. Unfortunately, establishing general sequence‐stability relationships by collecting experimental data from various studies is often restricted by extensive polymorphism as well as G4 structural and conformational transitions depending on the outer environment. As a consequence, the interpretation of thermodynamic data from experiment is often complicated. However, the global analysis of a wide variety of calorimetric and spectroscopic data acquired under different conditions promises to be a powerful approach for predicting both quadruplex folding and stability as a function of temperature, salt concentration, and co‐solutes. Also, biomolecular simulation methods may complement experiments and aid in an unbiased comparison of stabilities among different G4 structural isomers. Currently, our understanding of structure‐stability relationships and the impact of additional tertiary interactions, often involving different non‐adjacent domains within the primary sequence, is still limited. However, with a growing number of thermodynamic data, major principles of G4 stabilities become more and more apparent. It is thus anticipated that parameters determining the free energy of a quadruplex fold can better be assessed in the future to allow for a more reliable prediction of major G4 folds, associated with a better understanding of their interactions in a given environment.

## Conflict of interest

The authors declare no conflict of interest.

## Biographical Information

*Jagannath Jana obtained his MSc degree in Chemistry from Vidyasagar University (India). Following his PhD studies under the supervision of Dr. Subhrangsu Chatterjee at Bose Institute (Calcutta University, Kolkata, India), he was a postdoctoral researcher (2018‐2019) at Institut Curie (Paris, France) in the laboratory of Professor Stéphan Vagner. He is currently working as a postdoc with Prof. K. Weisz at the Institute of Biochemistry, Universität Greifswald (Germany). His research interests include thermodynamic and structural studies on G‐quadruplexes*.

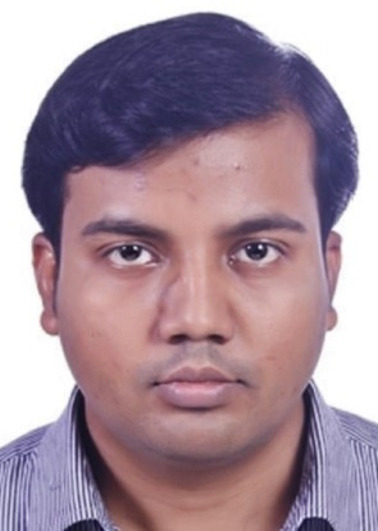

## Biographical Information

*Klaus Weisz received his MSc in Organic Chemistry (1983) as a DAAD fellow at the University of Cincinnati, Ohio (USA), and his Diploma in Chemistry (1985) at the University of Stuttgart (Germany). Following his doctoral studies at the University of Stuttgart (1990) he was a postdoctoral fellow at the University of California, San Francisco (1990‐1993) and a research fellow at the Free University of Berlin (1993‐2001), completing his habilitation in Physical Chemistry in 2000. Since 2001 he has been a Professor of Analytical Biochemistry at the Institute of Biochemistry, Universität Greifswald (Germany). His research focuses on nucleic acids with special emphasis on their tetra‐stranded structures*.